# Mathematical modeling and impact analysis of the use of COVID Alert SA app

**DOI:** 10.3934/publichealth.2022009

**Published:** 2021-11-29

**Authors:** Musyoka Kinyili, Justin B Munyakazi, Abdulaziz YA Mukhtar

**Affiliations:** Department of Mathematics and Applied Mathematics, Faculty of Natural Sciences, University of the Western Cape, Private Bag X17 Bellville 7535, South Africa

**Keywords:** COVID-19, COVID Alert SA app, digital-contact-tracing technology, compartmental deterministic models, mathematical modeling, mitigation measures

## Abstract

The human life-threatening novel Severe Acute Respiratory Syndrome Corona-virus-2 (SARS-CoV-2) has lasted for over a year escalating and posing simultaneous anxiety day-by-day globally since its first report in the late December 2019. The scientific arena has been kept animated via continuous investigations in an effort to understand the spread dynamics and the impact of various mitigation measures to keep this pandemic diminished. Despite a lot of research works having been accomplished this far, the pandemic is still deep-rooted in many regions worldwide signaling for more scientific investigations. This study joins the field by developing a modified SEIR (Susceptible-Exposed-Infectious-Removed) compartmental deterministic model whose key distinct feature is the incorporation of the COVID Alert SA app use by the general public in prolific intention to control the spread of the epidemic. Validation of the model is performed by fitting the model to the Republic of South Africa's COVID-19 cases reported data using the Maximum Likelihood Estimation algorithm implemented in fitR package. The model's sensitivity analysis and simulations stipulate that gradual to complete use of the app would be perfect in contact tracing and substantially reduce the plateau number of COVID-19 infections. This would consequentially contribute remarkably to the eradication of the SARS-CoV-2 over time. Proportional amalgamation of the app use and test for COVID-19 on individuals not using the app would also reduce the peak number of infections apart from the 50 – 50% ratio which spikes the plateau number beyond any other proportion. The study establishes that at least 30% implementation of the app use with gradual increase in tests conducted for individuals not using the app would suffice to stabilize the disease free equilibrium resulting to gradual eradication of the pandemic.

## 1. Introduction

In the late December 2019, strange respiratory complications in the form of pneumonia cluster cases were reported in Wuhan, the capital city of Hubei province of China [1]–[3]. Rigorous scientific investigations revealed that SARS-CoV-2 (Severe Acute Respiratory Syndrome Corona-virus-2) was the causative agent of the disease [1],[4]–[8]. The World Health Organization (WHO) recognized the virus as the cause of the disease on February 11, 2020 [9]. Based on the year of emergence, the new disease was finally named COVID-19 [10],[11]. Shortly after the first case of COVID-19 was reported, the disease mushroomed within most regions of the world posing life threats to humans [12]. Owing to its escalation, the World Health Organization (WHO) declared the disease a Public Health Emergency of international concern on January 30, 2020 and a global pandemic on March 11, 2020 [1],[13]. By the latter date, the total COVID-19 positive confirmed cases globally were 7,818 with over 1,370 severe cases and 170 deaths [14]. As of April 12, 2020, WHO confirmed 1,700,000 COVID-19 cases with over 105,000 fatalities globally [15]. By July 20, 2020, the disease had spread to more than 215 countries world-wide causing over 14.5 Million infections and more than 606,000 deaths [5]. The cumulative number of positive cases hit 35,220,166 with 1,037,604 demises in the entire globe by October 5, 2020 [14].

Reports in the literature divulge that the disease spilled over to humans from animals (specifically bats) followed by human-to-human transmission [5],[16]. The transmission of the virus among humans has been reported to be mainly via respiratory droplets produced during coughing, sneezing, talking and/or breathing of a COVID-19 infectious individual [2],[5],[8],[14],[17]. COVID-19 patients present respiratory symptoms which can be severe or mild [1],[4]. The common clinical symptoms reported include shortness of breath, dry cough, fever and severe pneumonia [1],[11],[18].

Following the outbreak of the pandemic, the fact that the disease has no cure and no vaccine that had been discovered by then, the World Health Organization was in the fore-front proposing several non-pharmaceutical preventive measures to be practiced by the public across the world in an effort for curtailing the spread of the SARS-CoV-2. These measures were not limited to washing of hands regularly with soap and water or cleaning them with alcohol-based hand rub, maintaining at least one-meter distance from people coughing or sneezing, avoiding touching one's face, staying at home if one feels unwell, covering one's mouth and nose during coughing or sneezing, refraining from smoking and other activities that weaken the lungs, practicing physical distancing by avoiding unnecessary travel, staying away from large groups of people, and wearing face masks. At the individual nation level, the governments of different countries in collaboration with their respective health departments and/or ministries, worked together to put in place and implement stringent measures in endeavor to contain the spread of the virus [19].

In Africa, the first COVID-19 positive case was reported on February 14, 2020 in Cairo the capital of Egypt by the Ministry of Health and Population [1],[13],[20]. In South Africa, the National Institute for Communicable Diseases (NICD) confirmed the first COVID-19 positive case on March 5, 2020 [2],[5],[13],[14],[21]. As of March 17, 2020, South Africa had 85 active cases for COVID-19 [2]. Due to the significant daily increase in the number of COVID-19 positive cases in the period between 5th and 15th march 2020, the government of the Republic of South Africa declared a national state of disaster to contain the spread of the SARS-CoV-2 [1],[2],[13],[22]. This pronouncement was followed by stringent measures not limited to travel restrictions, closure of learning institutions and lock-downs [21],[23]. Since April 12, 2020, South Africa had been leading with the top numbers of COVID-19 positive cases and hence becoming the foremost COVID-19 core in the entire continent of Africa [13]. According to the department of Health of the Republic of South Africa online resource and news portal on COVID-19 [24], the total confirmed COVID-19 cases were 1,149,591 with 31,368 fatalities as of January 6, 2021 and as of March 11, 2021, the country confirmed 1,525,648 cumulative cases with 51,110 fatalities.

Into the bargain of common non-pharmaceutical measures put in place in the fight against the epidemic, the information and communication technology (ICT) sector has been incorporated via development of android applications (apps) which work under different mechanisms and requirements. This technological pathway was firstly adopted in China and several other countries in East and South East Asia. It was found to be really successful in reducing the spread of the SARS-Cov-2 since it greatly simplifies contact tracing exercise [27]. A study done in [28] gives a comprehensive analysis of the digital contact-tracing solutions based on their methodologies and technologies in the light of data emerging about international experiences for deployment of digital contact-tracing pathway. More works exploring the subject covering the usage of the digital contact-tracing technological pathway employing different methodological approaches are also done in [29]–[36]. The technique is based on either data, WiFi or Bluetooth-contact tracing technology. In this belt, South Africa has not been left behind. Through the department of Health, COVID Alert SA app was devised. Guidelines from the Republic of South Africa department of Health online resource and news portal on COVID-19 [24] indicate that COVID Alert SA app is a South Africa's free exposure notification application via Bluetooth. It notifies people when they have been in close contact with someone who tested positive for COVID-19. To enable the usage of the app, one needs to download and install it in a device that enables Bluetooth connection such as smartphone. The application is wholly anonymous in that it preserves the user's confidentiality and security perpetually. The app does not even require or reserve any personal information. In the event of exposure notification, neither personal information is displayed nor disclosed to the person being notified. The usage of the app complies with the socio-technical framework for digital contact tracing annotated in [37].

On the strength of the fact that SARS-Cov-2 is a pressing threat to human life, it promptly captured the attention of researchers globally since its first report in Wuhan, China. This evoked the investigations in the scientific arena to be kept activated. The insight has been to understand the spread dynamics and control measures of the epidemic. Mathematical modeling has greatly been utilized in this circumstance [38]–[41].

Inference by [5] confirms that mathematical modeling is a utility tool for providing realistic discernment into the transmission dynamics and control of quickly spreading infectious disease such as COVID-19. Modified SIR (Susceptible-Infected-Recovery/Removed) and SEIR (Susceptible-Exposed-Infected-Recovery/Removed) compartmental models have been mostly put in use. For instance in [39], a modified SEIR mathematical model was developed to investigate the transmission dynamics of the COVID-19 epidemic. Similar works on the transmission dynamics of the pandemic using various devised models were also done in [5],[6],[13],[16],[17],[20],[38],[41]–[44]. A study in [45] used a modified SEIR compartmental model to study the effects of social distancing as an intervention measure against the spread of the COVID-19 epidemic. Modeling the effects of social distancing in conjunction with other mitigation measures to curtail the spread of the SARS-CoV-2 was also done in [2],[4],[5],[19],[46]. The work done in [47] used mathematical modeling to assess impact of use of face masks with other control measures in an effort to control the spread of the pandemic. Similar modeling works on the effectiveness of use of face masks to control the spread of COVID-19 were also done in [10],[12],[48]–[50]. An hybrid model predictive controller to optimize lock-down management under different scenarios was used in [23].

Despite many investigations on the pandemic being accomplished, the epidemic is still deep-rooted in many regions worldwide including South Africa. This calls for more scientific research to be conducted. In light of this, this study sought to develop a compartmental deterministic model specifically to assess the potential impact of using COVID Alert SA application by the general public in an effort to break the chain of transmission of the novel COVID-19 epidemic and simplify the contact tracing exercise.

The rest of the paper is organized as follows: The model is developed and fitted in Section 2 as parts of materials and methods. In Section 3, we prove the positivity and boundedness of the solution as well as the stability properties of the model. Section 4 is devoted to numerical results and discussion pertaining to sensitivity analysis and simulations. Finally, Section 5 gives the conclusion

## 2. Materials and methods

### 2.1. Model development

We begin by describing and formulating the proposed deterministic model incorporating the use of the COVID Alert SA app. Let the total human population at time *t* be denoted by *N*(*t*). This total population is hereby subdivided into six classes which are mutually exclusive namely the susceptible class (*S*(*t*)), the exposed class of individuals who are using the COVID Alert SA app (*E_A_*(*t*)), the exposed class of individuals who are not using the app (*E*(*t*)), the class of individuals who are tested for COVID-19 regardless of whether using the app or not (*T*(*t*)), the class of infectious individuals (*I*(*t*)) and the class of the individuals who have been removed from the chain of transmission of the disease (*R*(*t*)). Therefore, the cumulative human population *N*(*t*) for the proposed model is given by

$$N(t)=S(t)+E_{A}(t)+E(t)+T(t)+I(t)+R(t).$$ (1)

**Figure 1.**
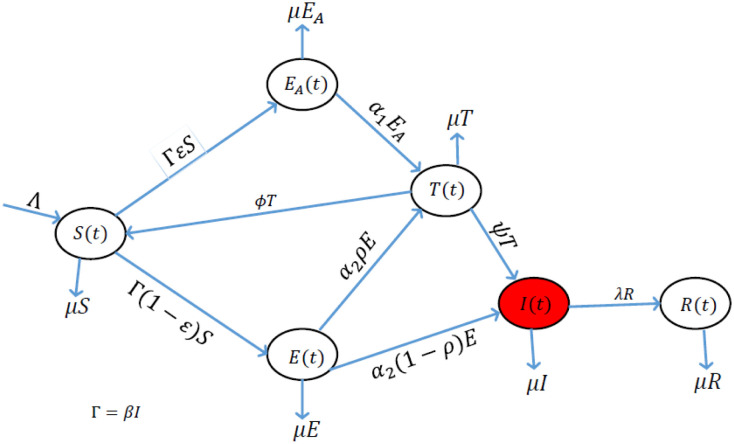
The proposed model incorporating the use of COVID Alert SA app.

The flow diagram for the proposed model is represented by Figure 1. This model is an extension of the standard SEIR model used widely to study the spread and transmission dynamics of COVID-19. We added the compartment for individuals using the COVID Alert SA app in line with the functioning of the app as aforementioned in Section 1 and elaborated in [24]. We also added the compartment for individuals who are tested against the COVID-19.

Basically, the model shows that the susceptible individuals can move to the class of individuals who are using the app denoted by *E_A_*(*t*) or progress to the normal exposed class of individuals who are not using the app denoted *E*(*t*). We stress that the *E_A_*(*t*) class accounts for the collection of all the individuals who receive exposure notifications via the app pertaining their close contacts with anyone who tested positive for COVID-19. Whereas the *E*(*t*) class collects all the individuals who do not receive any exposure notification since they are not using the app. The individuals using the app progress immediately for a test (at the rate of *α*_1_) to confirm their COVID-19 status on receiving the exposure notifications. The immediate logical decision to go for a test upon exposure notification is articulated to the assumption that, since COVID-19 is life-threatening and people fear succumbing to the disease, one moves for a test without hesitation on exposure notification. Also, the possible stigmatization attached to the infected and/or recovered individuals dictates the behaviour change of individuals who receive exposure notifications. Since individuals who are not using the app do not receive any exposure notification, they either get tested at the rate of *α*_2_*ρ* or progress directly to the infectious compartment at the rate of *α*_2_(1 − *ρ*). The assumptions here are that, individuals who are not using the app progress for a test by either volunteering or by normal manual contact-tracing probably by the government or by making efforts to meet some conditions such as traveling abroad where a COVID-19 negative certificate is a requirement, or by any other valid reasons. The other individuals not using the app notice their COVID-19 infection status when symptoms start appearing hence progressing directly to the infectious class. The infectious class (*I*(*t*)) in this model collects all the individuals who are capable of shedding the virus regardless of whether symptomatic or asymptomatic. The tested individuals can be positive or negative against the disease. If they test positive, they progress to the infectious class at the rate of *ψ*. We assume here that individuals who become positive after undergoing the COVID-19 test, are immediately isolated, quarantined and/or hospitalized and hence do not contribute in spreading the virus. Individuals who test negative are assumed to go back to the susceptible class at the rate of *φ*. We make this assumption since it is clear that even with the control measures put in place, there is a chance of contracting the virus especially with the situation of the divergent COVID-19 variants being witnessed. Finally, the infectious individuals get removed from the chain of transmission of the disease at the rate of *λ*. The class *R*(*t*) collects all the individuals who are removed from the transmission chain of the disease. These individuals include those who have recovered from the COVID-19 infections and individuals who are deceased due to the COVID-19 [51].

Thus the consequential model equations are

$$\frac{dS}{dt}=\Lambda +\varphi T-(\Gamma +µ)S,$$ (2)

$$\frac{dE_{A}}{dt}=\Gamma \varepsilon S-(\alpha_{1}+µ)E_{A},$$ (3)

$$\frac{dE}{dt}=\Gamma (1-\varepsilon )S-(\alpha_{2}+µ)E,$$ (4)

$$\frac{dT}{dt}=\alpha_{1}E_{A}+\alpha_{2}\rho E-(\psi +\varphi +µ)T,$$ (5)

$$\frac{dI}{dt}=\psi T+\alpha_{2}(1-\rho )E-(\lambda +µ)I,$$ (6)

$$\frac{dR}{dt}=\lambda I-µR.$$ (7)

The term Γ=βI is the force of infection in the model.

The model parameters and the state variables are defined to be positive since the model monitors human population. The parameters used in the model are listed in Table 1. Considering the fact that several modeling research works on COVID-19 dynamics have been done before our work, we obtained some parameter values that exist in the literature and estimated others that do not exist in line with our proposed model.

**Table 1.** Parameter description for the model (2)–(7) and their estimated values.

Symbol	Parameter Description	Value per day	Source
Λ	Recruitment rate	11244	[2], [41]
*β*	Effective contact rate	1.1411	[2]
*ε*	Measure of the COVID Alert SA app use	(0,1)	Variable
*α*_1_	Rate of test for the app users who receive exposure notifications	0.99	Assumed
*α*_2_*ρ*(*α*_2_(1 − *ρ*))	Rate of test (infection) for the exposed individuals not using the app	*α*_2_ = 0.6	[25]
*ρ*	Measure of test for exposed individuals not using the app	(0,1)	Variable
*φ*	Rate of susceptibility by the tested individuals (Only for those individuals who test negative for the COVID-19)	0.5	Fitted
*ψ*	Infection rate by the tested individuals	0.232	Fitted
*λ*	Rate of removal from transmission chain	0.2109	[25], [26]
*µ*	Natural death rate	0.0001	[41]

### 2.2. Model fitting

**Figure 2.**
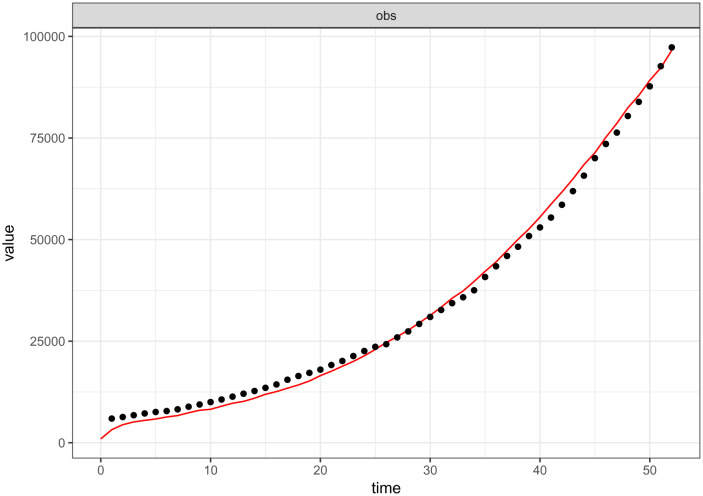
The model (2)–(7) fit to cumulative COVID-19 positive reported cases (1 May–21 June 2020) in South Africa. The black-dotted line represents the reported data for COVID-19 positive cases while the red-continuous line shows the goodness of fit. The parameter values used are as listed in Table 1, *ε* = 0.001 and *ρ* = 0.9.

Fitting of any formulated model to data is a golden key aspect especially for the validation of the model. This warrants and authorizes the use of the model for the intended purpose. On account of this, the model (2)–(7) was fitted to the cumulative COVID-19 positive cases data as reported by the government of the Republic of South Africa in the period between 1st of May 2020 and 21st of June 2020 via [24]. The Maximum Likelihood Estimation (MLE) algorithm implemented in fitR package was adopted to fit the model to the data. The fitted model is shown in Figure 2.

## 3. Analytical results

Since the model describes human population, analysis of the model's properties is very key especially in preserving the epidemiological meaningfulness [41], [52]. Due to this paramount reason, we analyze the properties of the model system (2)–(7).

### 3.1. Positivity of the solution

We investigate if the model solutions remain positive for all *t* ≥ 0. This is done by stating and proving a theorem as follows:

***Theorem 3.1***

Let *S*(0) > 0, *E_A_*(0) > 0, *E*(0) > 0, *T*(0) > 0, *I*(0) > 0 and *R*(0) > 0. Then *S*(*t*) > 0, *E_A_*(*t*) > 0, *E*(*t*) > 0, *T*(*t*) > 0, *I*(*t*) > 0 and *R*(*t*) > 0, ∀*t* ≥ 0.

***Proof***

Suppose that the solution of the model equations (2)–(7) is not positive for all *t* ≥ 0. Then there exist a first time $\tilde{t}>0$ such that

$$\tilde{t}=inf\{t|S(t)=0\;or\;E_{A}(t)=0\;or\;E(t)=0\;or\;T(t)=0\;or\;I(t)=0\;or\;R(t)=0\}.$$

If $S(\tilde{t})=0$, then ∀ $t\in (0,\tilde{t})$, *S*(*t*) > 0, *E_A_*(*t*) > 0, *E*(*t*) > 0, *T*(*t*) > 0, *I*(*t*) > 0 and *R*(*t*) > 0, $\frac{dS(\tilde{t})}{dt}<0$. However, from (2) $\frac{dS(\tilde{t})}{dt}=\Lambda +\varphi T(\tilde{t})>0$ (since all the model parameters are defined to be positive) which contradicts the initial assumption that $\frac{dS(\tilde{t})}{dt}<0$. This clearly depicts that *S*(*t*) > 0. Arguing similarly, it can be established that *E_A_*(*t*), *E*(*t*), *T*(*t*), *I*(*t*) and *R*(*t*) for the model equations are positive for all *t* ≥ 0.

### 3.2. The invariant region

We next investigate if the model solutions are bounded and remain in the positive region for all *t* ≥ 0. This is done by proving that the biological feasible region predefined here as

$${𝒥}_{ℜ}=\left\{\left(S,E_{A},E,T,I,R\right)\in {\mathbb{R}}_{+}^{6}:S+E_{A}+E+T+I+R\leq \frac{\Lambda }{µ}\right\},$$ (8)

is positively invariant with respect to the model (2)–(7). Getting the time derivative of the total population *N*(*t*), we obtain

$$\frac{dN}{dt}=\Lambda -µN.$$ (9)

By separation of variables and integration factor techniques, we have

$$N(t)=\frac{\Lambda }{µ}\left(1-e^{-µt}\right)+N_{0}e^{-µt},$$ (10)

where *N*_0_ = *N*(0).

Using comparison theorems on ODEs (10) (see [53]) yields

$$\underset{t\to \infty }{lim}N(t)=\frac{\Lambda }{µ}.$$ (11)

Since *N*(*t*) is a monotonically increasing function, if $N(0)\leq \frac{\Lambda }{µ}$ then $N\leq \frac{\Lambda }{µ}$, ∀*t* ≥ 0. This infers that *N* is bounded thus we conclude that the feasible region 𝒥*_ℜ_* is positively invariant and attracting. Hence it is worthwhile considering the dynamics of the model (2)–(7) in 𝒥*_ℜ_* for all *t* ≥ 0. The model can now be considered as epidemiologically and mathematically well posed in 𝒥*_ℜ_* [54].

### 3.3. Stability analysis

We analyze the stability of the model's disease free equilibrium (DFE). In the DFE situation, it stipulates that no infectious individual in the population under investigation. We denote the model's DFE point by *ℰ*_0_ and define as

$${ℰ}_{0}=\left(\frac{\Lambda }{µ},0,0,0,0,0\right).$$ (12)

#### 3.3.1. Local stability of the disease free equilibrium

To determine the local stability of the DFE, we compute the basic reproduction number of the model which we denote by *ℜ*_0_. It represents the average secondary number of infections that result from one infectious individual when introduced in a totally susceptible population [41]. We adapt the next generation matrix (NGM) method to find the value of the *ℜ*_0_ [52]. From the model (2)–(7) the secondary infections ℱ and the transfer of infections 𝒱 are respectively given by

$$ℱ=\left(\begin{array}{c} \beta \varepsilon IS\\ \beta (1-\varepsilon )IS\\ 0\\ 0 \end{array}\right)$$ (13)

and

$$𝒱=\left(\begin{array}{c} (\alpha_{1}+µ)E_{A}\\ (\alpha_{2}+µ)E\\ -\alpha_{1}E_{A}-\alpha_{2}\rho E+(\psi +\varphi +µ)T\\ -\psi T-\alpha_{2}(1-\rho )E+(\lambda +µ)I \end{array}\right).$$ (14)

Thus,

$$F=\left(\begin{array}{cccc} 0 & 0 & 0 & \beta \varepsilon \frac{\Lambda }{µ}\\ 0 & 0 & 0 & \beta (1-\varepsilon )\frac{\Lambda }{µ}\\ 0 & 0 & 0 & 0\\ 0 & 0 & 0 & 0 \end{array}\right)$$ (15)

and

$$V=\left(\begin{array}{cccc} \alpha_{1}+µ & 0 & 0 & 0\\ 0 & \alpha_{2}+µ & 0 & 0\\ -\alpha_{1} & -\alpha_{2}\rho & \psi +\varphi +µ & 0\\ 0 & -\alpha_{2}(1-\rho ) & -\psi & \lambda +µ \end{array}\right).$$ (16)

$$FV^{-1}=\left(\begin{array}{cccc} \frac{\beta \varepsilon \alpha_{1}\psi \Lambda }{𝒦ℳ𝒲µ} & \frac{\beta \varepsilon \alpha_{2}\left(\rho \psi +(1-\rho )ℳ\right)\Lambda }{ℒℳ𝒲µ} & \frac{\psi \beta \varepsilon \Lambda }{ℳ𝒲µ} & \frac{\psi \beta \varepsilon \Lambda }{𝒲µ}\\ \frac{\beta (1-\varepsilon )\alpha_{1}\psi \Lambda }{𝒦ℳ𝒲µ} & \frac{\beta (1-\varepsilon )\alpha_{2}\left(\rho \psi +(1-\rho )ℳ\right)\Lambda }{ℒℳ𝒲µ} & \frac{\psi \beta (1-\varepsilon )\Lambda }{ℳ𝒲µ} & \frac{\psi \beta (1-\varepsilon )\Lambda }{𝒲µ}\\ 0 & 0 & 0 & 0\\ 0 & 0 & 0 & 0 \end{array}\right),$$ (17)

where

$$𝒦=\alpha_{1}+µ,$$

$$ℒ=\alpha_{2}+µ,$$

ℳ=ψ+φ+µ,

𝒲=λ+µ.

Hence using Octave software, the basic reproduction number for the model is

$${ℜ}_{0}=\frac{\beta \Lambda }{𝒲µ}\frac{\alpha_{2}(1-\varepsilon )\left(\rho \psi +(1-\rho )ℳ\right)𝒦+\alpha_{1}\psi \varepsilon ℒ}{𝒦ℒℳ}-\frac{\beta \Lambda }{𝒲µ}\sqrt{\frac{\alpha_{1}\alpha_{2}\psi \varepsilon (1-\varepsilon )\left(\rho \psi +(1-\rho )ℳ\right)}{𝒦ℒℳ}\left(\frac{1-ℳ}{ℳ}\right)}.$$ (18)

The DFE of our model is locally asymptotically stable if *ℜ*_0_ < 1 and unstable if *ℜ*_0_ > 1.

#### 3.3.2. Global Stability of the Disease Free Equilibrium

To investigate the global stability of the DFE for the model, we adapt the method used by [55]. A theorem in this case is stated and proven in Appendix.

## 4. Numerical results and discussion

### 4.1. Sensitivity analysis

Generally, sensitivity analysis enables a researcher to ascertain the effect(s) of certain parameter(s) of research interest on the dependent variable [41]. For our model, we carry out the sensitivity analysis to determine the impact(s) of the use of the COVID Alert SA app hereby measured by the parameter *ε* (the chief parameter of interest for this study) and the testing for COVID-19 (for individuals not using the app) measured by the parameter *ρ* on the basic reproduction number *ℜ*_0_. We define to have perfect app use and perfect testing against COVID-19 for individuals not using the app at the instance when *ε* and *ρ* respectively draw near unity whereas poor app use and testing is encountered when both the parameters tend to zero, thus 0 < *ε* < 1 and 0 < *ρ* < 1. The sensitivity analysis is hereby done graphically using (18).

**Figure 3.**
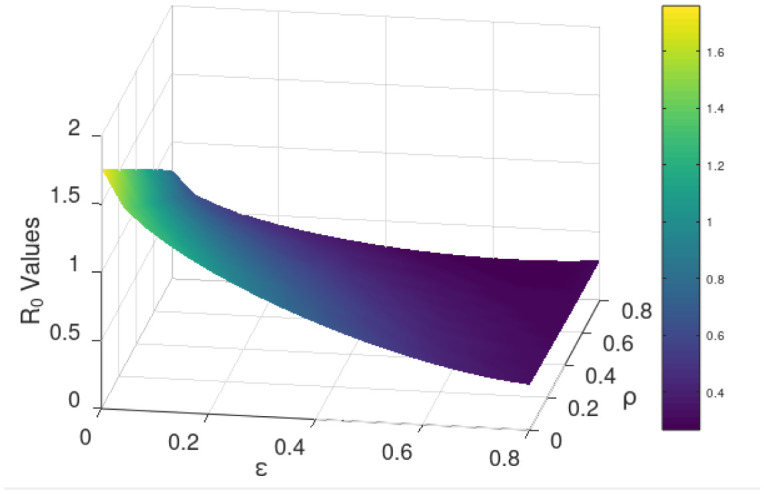
Variation of the basic reproduction number with the measure of the use of COVID Alert SA app *ε* and measure of test for COVID-19 *ρ* for exposed individuals not using the app. Parameter values used are listed in Table 1.

**Figure 4.**
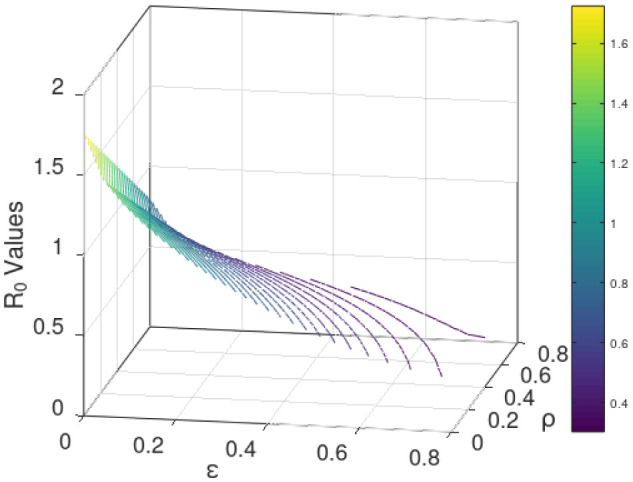
Contour plot visualization for Figure 3.

Figure 3/Figure 4 establishes how the basic reproduction number varies with the measure of the use of COVID Alert SA app *ε* and measure of test for COVID-19 *ρ* for exposed individuals not using the app. The figure vividly hints that both the parameters *ε* and *ρ* are inversely proportional to *ℜ*_0_. However, distinctively it obliques that *ℜ*_0_ decreases more gently drawing close to 0 as *ε* assumes its extreme value close to unity (perfect use of the app). The figure also indicates that although *ℜ*_0_ reduces with increase in *ρ*, the *ℜ*_0_ value still remains higher almost close to unity. This stipulates that test against the COVID-19 for exposed individuals only without use of the app may not fast and completely diminish the spread of the pandemic. This is probably because without the use of the app, no exposure notifications and thus contact-tracing exercise is manually done allowing chances of missing some unknown exposures who drive more infections. This therefore calls for blending testing for COVID-19 with use of the app. This solitary trend fundamentally divulges that the increased implementation of the use of the COVID Alert SA app by the general public in presence of testing, would greatly diminish the basic reproduction number and thus the DFE being stabilized to the extend of the pandemic eradication over time.

**Figure 5.**
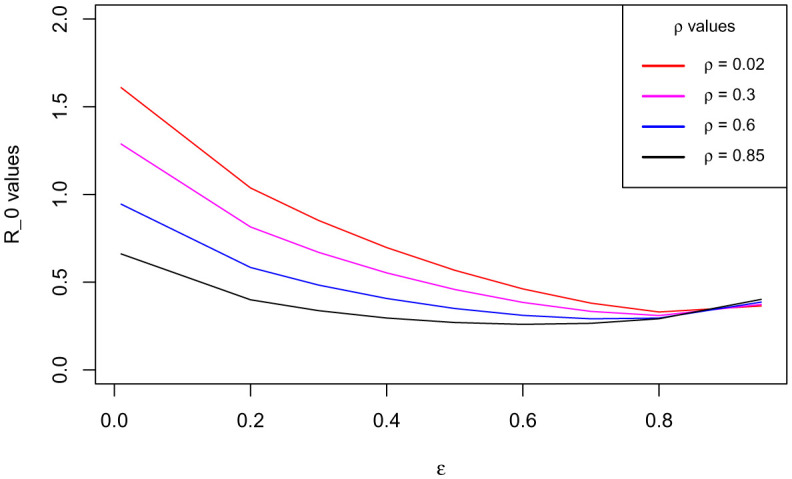
Variation of the basic reproduction number with the measure of the use of COVID Alert SA app *ε* at rising values of measure of test for COVID-19 *ρ*.

**Figure 6.**
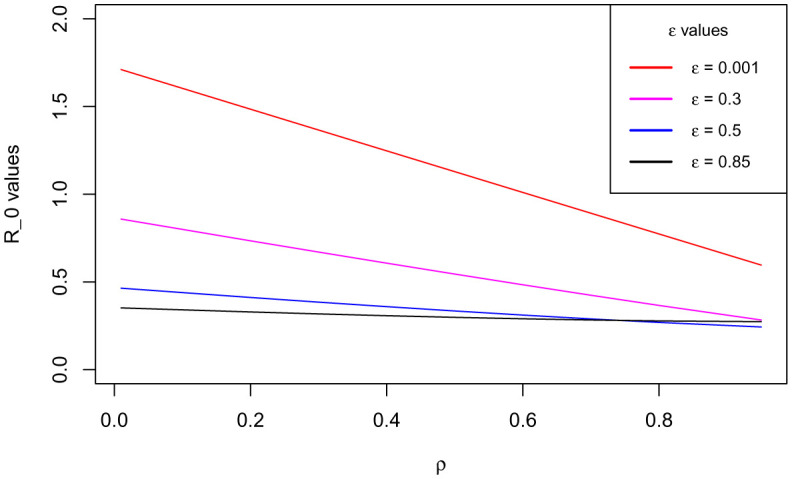
Variation of the basic reproduction number with the measure of test for COVID-19 *ρ* at increasing values of the measure of the use of COVID Alert SA app *ε*.

Additionally, with the validation from Figure 3/Figure 4 that *ℜ*_0_ is inversely proportional to both *ε* and *ρ* and the fact that full implementation of any protocol takes time, we consider endorsing each parameter in bits. Figure 5 shows how *ℜ*_0_ grows small as *ε* advances near its extreme value 1 at rising values of *ρ*. Figure 6 illustrates how *ℜ*_0_ declines as *ρ* proceeds towards its maximum value 1 at increasing values of *ε*. The two figures plainly reveal the shift of *ℜ*_0_ values as both parameters draw near their utmost value (unity) where we have perfect use of the app and testing for the disease. Uniquely, it is observed that increasing the value of *ε* accelerates the shift of the *ℜ*_0_ values in presence of testing. This discloses that if the use of the COVID Alert SA app by the general public, would increasingly be implemented concurrently with testing for the COVID-19, then this would remarkably diminish the basic reproduction number. This would lead to the DFE becoming stable and consequentially getting rid of the COVID-19 pandemic over relatively shorter time. Crucially, Figure 6 distinctively divulges that implementing at least 30% usage of the app simultaneously alongside gradually increasing measure of test for individuals not using the app would be sufficient to stabilize the DFE.

This fundamental discovery is absolutely in line with the functioning of the app and the logical behavior of individuals on exposure notification. The model assumes that due to the fact that the COVID-19 pandemic is life threatening and no one who wishes and admires to contract the disease, then sensibly once the people using the app get exposure notifications, they take immediate action to voluntarily present themselves for a COVID-19 test. On the event that one is not using the app, then mass and volunteer testing is carried out increasing the measure for testing. This combined behavior change can accelerate the measure for testing and hence running synchronously with the use of the app. This can ultimately bring the basic reproduction to less than unity hence eliminating the epidemic. Stipulation from this consequence, discloses the novelty of the proposed model.

### 4.2. Simulations

We use the fitted model to carry out simulations for testing of different scenarios with respect to the parameter of interest. We firstly consider varying use of the app with constant testing for individuals not using the app and secondly consider endorsing use of the app and testing for people not using the app proportionally. This is because in reality, it is perhaps hard to achieve perfect use of the app and perfect testing without app use. The parameter values used are as listed in Table 1.

**Figure 7.**
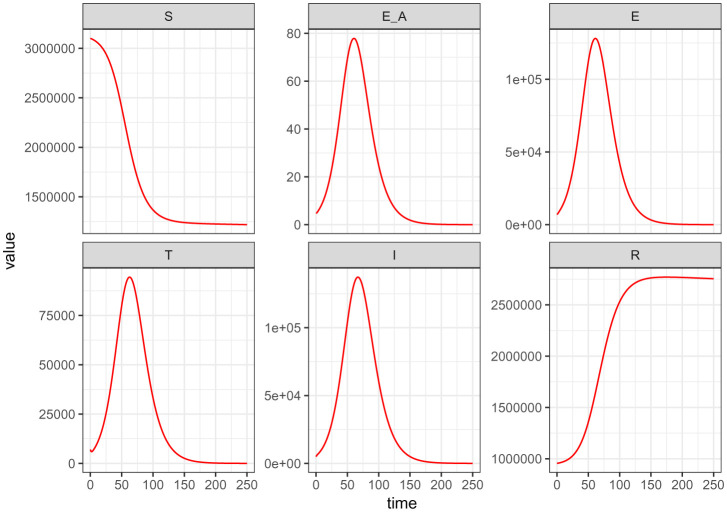
Simulations of the model (2) – (7) showing the trajectory for all the model classes when the measure for the use of the COVID Alert app *ε* is at its extreme minimum value.

Figure 7 illustrates the trajectory of all the model (2) – (7) classes at the instant when the measure for the use of the COVID Alert app *ε* is at its extreme minimum value. This figure clearly predicts higher plateau number of exposures and consequentially higher infections. This is because without the use of COVID Alert SA app, there is no exposure notifications and thus this paves way for more unknown exposures surging the peak number of the cumulative infections.

**Figure 8.**
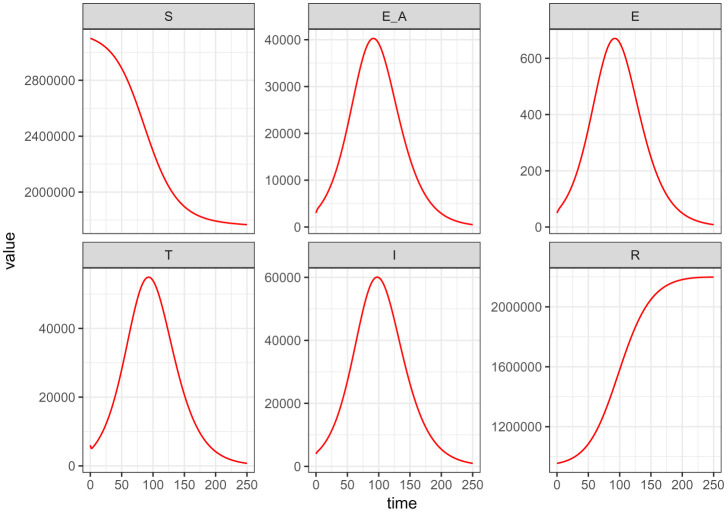
Simulations of the model (2) – (7) showing the trajectory for all the model classes when the measure for the use of the COVID Alert app *ε* is at its extreme maximum value.

Figure 8 depicts the trajectory of all the model (2) – (7) classes at the instant when the measure for the use of the COVID Alert app *ε* is at its extreme maximum value. This Figure plainly establishes higher plateau number of exposure notifications (perfect contact tracing), low number of unknown exposures and diminishing peak number of infections as a result. This is because with the use of COVID Alert SA app, all exposures will be notified leading to no unknown exposures and hence declining the cumulative infections.

**Figure 9.**
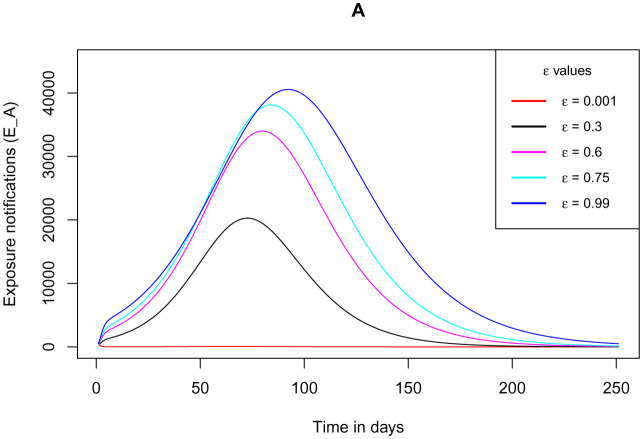
Simulations of the model (2) – (7) showing cumulative number of exposed individuals (exposure notifications) using the COVID Alert SA as the parameter *ε* for the measure of use of the app varies.

Figure 9 demonstrates how the cumulative number of exposed individuals (exposure notifications) who are using the COVID Alert SA varies as the parameter *ε* for the measure of use of the app increases from its minimum value to its maximum value unity. It indicates that as the measure of the use of the app increases, the peak for the cumulative number of the exposure notifications increases. This is absolutely in line with the functioning of the app in exposure notifications on the close contacts with individual(s) who tested positive for the disease. This number is expected to increase due to the Bluetooth-contact-tracing technology facilitated by the app. A Suggestion from this observation is that the app performs the best in contact tracing exercise making it simple as opposed to the manual contact tracing which has a high probability of missing several close contacts. These missed close contacts make the COVID-19 positive cases to spike implying that with the use of the app, the pandemic can easily be contained.

**Figure 10.**
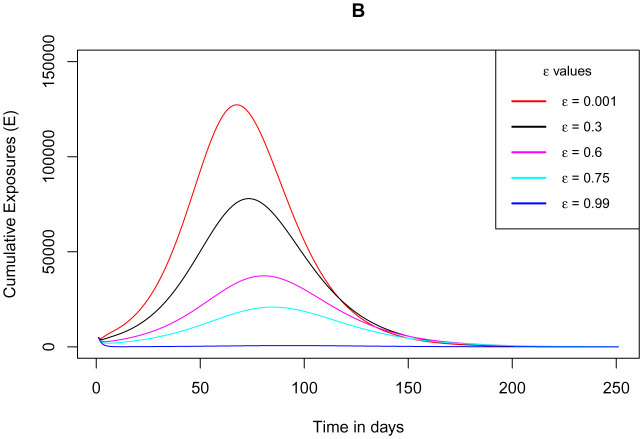
Simulations of the model (2) – (7) showing cumulative number of exposed individuals not using the COVID Alert SA as the parameter *ε* for the measure of use of the app varies.

Figure 10 shows how the cumulative number of exposed individuals who are not using the COVID Alert SA varies as the parameter *ε* for the measure of use of the app draws near its extreme value 1. The figure indicates that the total number of exposed individuals not using the app reduces as the measure of the use of the app rises close to 1. This stipulation is entirely in concurrence with the functioning of the app since as more people start using the app, the number of people not using the app from the susceptible population would as well decrease. At the instant when all the individuals from the susceptible population are using the app, then there would be no exposed individuals not using the app. This could be the best instance because the use of the app would have been implemented 100% and thus any exposure to the infection would definitely be notified by the app. This would as well bring the cumulative number of infections down, since contact tracing would be 100% thus totally diminishing the unknown exposures.

**Figure 11.**
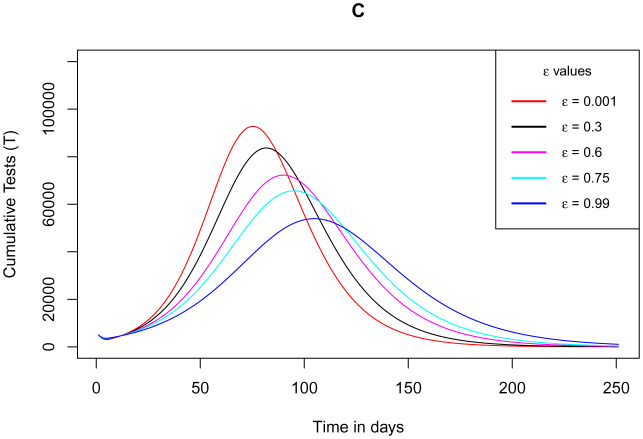
Simulations of the model (2) – (7) showing cumulative number of tested individuals against COVID-19 regardless of whether using the app or not as the parameter *ε* for the measure of use of the app varies.

Figure 11 illustrates how the cumulative number of tested individuals against COVID-19 (regardless of whether using the app or not) varies as the parameter *ε* for the measure of use of the app increases close to 1. It exhibits that as the measure of the use of the app tends to 1, the number of tests conducted diminish until it is almost equal to the cumulative number of exposure notifications. This is because the individuals using the app are sensibly assumed to present themselves for a COVID-19 test immediately after exposure notifications. Completely, this is in order since as more people start using the app, the exposed individuals not using the app simultaneously drop hence having no more people being tested from such class. This infers that test for COVID-19 is carried out after exposure notifications via the app in which in the simulation for the model, the rate of test after exposure notifications *α*_1_ is assigned its maximum value very close to unity. Refer to values in Table 1.

**Figure 12.**
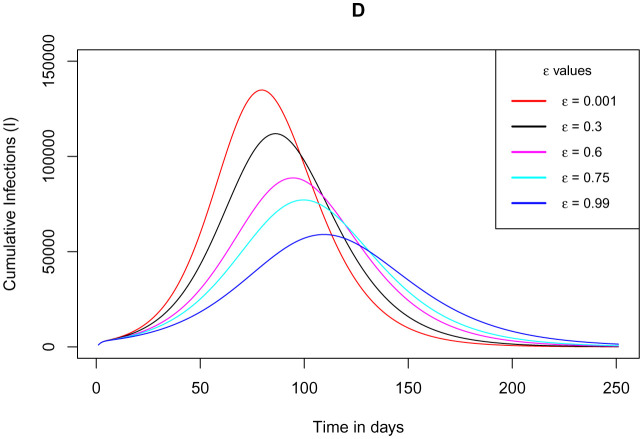
Simulations of the model (2) – (7) showing cumulative number of infections as the parameter *ε* for the measure of use of the app varies.

Figure 12 depicts how the cumulative number of infections varies as the parameter *ε* for the measure of use of the app gets larger close to its climax value unity. The figure manifests that, as the measure of the use of the app assumes its extreme value, the plateau number of cumulative infections declines. This indication is entirely in accordance with the expected outcome as long as the app is in use and performs its function accurately. The stipulation here, solely authenticates that serious adoption and implementation of the app use can gradually eradicate the pandemic over time.

**Figure 13.**
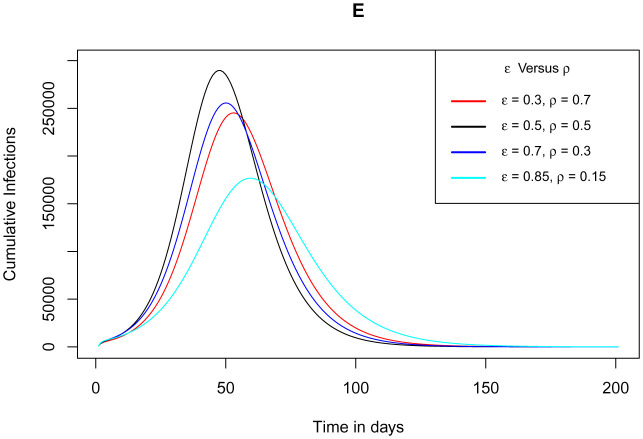
Simulations of the model (2) – (7) showing cumulative number of infected individuals as the parameter *ε* for the measure of use of the app and the parameter *ρ* for the measure of tests conducted for the exposed individuals not using the app vary proportionally.

Figure 13 establishes how the cumulative number of infections varies as the parameter *ε* for the measure of use of the app and the parameter *ρ* for the measure of tests conducted for the exposed individuals not using the app vary proportionally. The proportions used here are respectively for *ε* - *ρ* in 30 – 70%, 50 – 50%, 70 – 30% and 85 – 15%. For the ratios 30 – 70%, 70 – 30% and 85 – 15%, the plateau number for cumulative case infections declines gently though not completely. This is because for each ratio, there is always a proportion of exposed individuals who are not using the app and get infected directly before being tested and thus these individuals spread the SARS-CoV-2 unknowingly. However for the proportion 50 – 50%, the peak number of cumulative case infections spike beyond any other proportion. This is due and entirely valid since 50% of the exposed individuals not using the app, don't get tested and therefore they contract the virus only to notice that they are infected once symptoms start appearing. This is dangerous and thus it contributes to more unaware exposures rising the basic reproduction number, hence the new infections surging. This propounds that the good ratio to curtail the spread of the virus can be any of the aforementioned options apart from the 50 – 50% and as the use of the app proportion increases, the better since the number of infections keep on diminishing.

**Figure 14.**
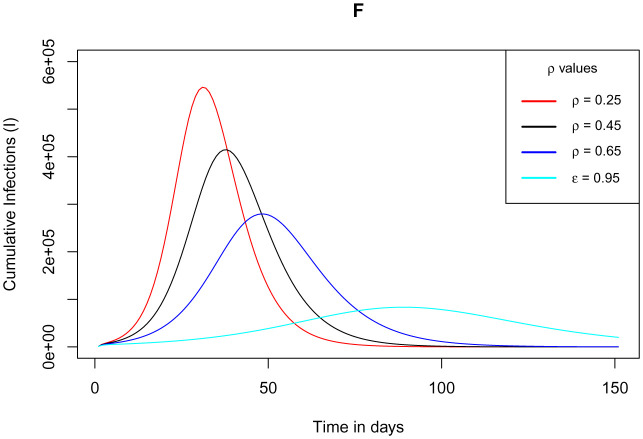
Simulations of the model (2) – (7) showing cumulative number of infected individuals at 30% use of the app *ε* and increasing measure of tests *ρ* conducted for the exposed individuals not using the app.

As verified by Figure 6, the DFE for the model stabilizes with at least 30% use of the app alongside gradually increasing measure of test for exposed individuals not using the app. This observation establishes the threshold for the app use. We simulate this scenario for the model. The simulation is validated by Figure 14, where it stipulates plainly that the plateau number of cumulative infections decline right from the measure of the app baseline value as the measure of test against the COVID-19 for exposed individuals not using the app increases. It is clearly observed that at low measure of test, the peak number of infections is high but it still takes a relatively shorter time for the infections to clear. Also, as the measure of test increases, the peak number of infections reduces but it is delayed hence the infections clear at a delayed time.

## 5. Conclusions

In this study, a modified SEIR deterministic model was developed. The paramount distinctive feature of the model was the assimilation of use of the COVID Alert SA app by the general public in an effort to shatter the chain of transmission for the novel COVID-19 pandemic. Analysis of the key model properties was carried out confirming that the model solution remained positive and bounded for all non-negative time.

Furthermore, the deterministic model was validated by fitting it to COVID-19 positive reported data of the Republic of South Africa. This authorized the model to be used for simulations. In the absence of the use of the app, the model predicted surging plateau numbers of unknown cumulative exposures and infections consequentially. Whereas in extreme value of use of the app, the model established perfect contact tracing, low number of unknown exposures and diminishing peak number of infections as a result.

Sensitivity analysis performed for the model revealed that the use of the app was inversely proportional to the basic reproduction number. This stipulation meant that gradual to full implementation of the app use by the general public would significantly accelerate the stabilization of the disease free equilibrium hence eradicating the epidemic over relatively shorter time. It was importantly established that, the threshold for the app use to be 30% with gradually increasing measure of test against COVID-19 for individuals not using the app. This consideration was made since not everyone in South Africa owns a smartphone.

In line with the functioning mechanism of the COVID Alert SA app, simulations disclosed as follows: The increased use of the app would work perfectly and simplify the contact tracing exercise. This was confirmed by the rising peak number of the individuals using the app as the measure of the app use drew near its maximum value 1. The number of the exposed individuals not using the app declined remarkably as the use of the app parameter approached its climax value unity. This aspect pre-emptied that the number of infections would synchronously drop with implementation of the app use. Fundamentally, the plateau number of the infected individuals diminished with the use of the app measure assuming its extreme value 1. This tendency absolutely stipulated that gradual to complete implementation of the app use would lead to elimination of the pandemic over time.

Owing to the fact that, implementing the use of the app is a gradual process and the fact that achieving perfect testing is a challenge, the model simulations considered amalgamating the use of the app *ε* and measure of test against COVID-19 for the exposed individuals not using the app *ρ*. The two parameters *ε* - *ρ* were respectively endorsed proportionally in 30 – 70%, 50 – 50%, 70 – 30% and 85 – 15%. The peak number of infections was found to decline in the ratios 30 – 70%, 70 – 30% and 85 – 15%. However, there was a spike in the plateau number of infections beyond any other proportion for the 50 – 50% option, since this ratio implied that 50% of the exposed individuals not using the app are not tested but notice their infections on appearance of symptoms. This greatly contributes to more unknown exposures and hence rise in new infections. Simulations at the 30% baseline use of the app with gradually increased testing for individuals not using the app showed high reduction in the peak number of infections at a delayed clearance of the epidemic. The study advocates for at least 30% implementation of the app use as long as it performs its intended function accurately alongside gradual increase in the testing capacity.
