# Supplementary material for: Mathematical modeling and impact analysis of the use of COVID Alert SA app
Source: AIMS Public Health. 2021 Nov 29;9(1):106–28. doi: 10.3934/publichealth.2022009 (PMC8755967; doi:10.3934/publichealth.2022009)
Supplement: Supplementary file 1 [file publichealth-09-01-009-s001.pdf]

Public Health

http://www.aimspress.com/journal/aimsph/

AIMS Public Health, 9(1): 106–128.
DOI:10.3934/publichealth.2022009
Received: 07 September 2021
Accepted: 23 November 2021
Published: 29 November 2021

Research article

Mathematical modeling and impact analysis of the use of COVID Alert SA app

Musyoka Kinyili *, Justin B Munyakazi and Abdulaziz YA Mukhtar

Department of Mathematics and Applied Mathematics, Faculty of Natural Sciences, University of the Western Cape, Private Bag X17 Bellville 7535, South Africa

* **Correspondence:** Email: 4100150@myuwc.ac.za, davismusyooo@gmail.com; Tel: +254710681113.

Appendix

1. Global stability of the disease free equilibrium

Theorem 3.3.2

Let the model be expressible in the form, $\frac{d\mathbf{P}}{dt} = F(\mathbf{P},\mathbf{Q})$, $\frac{d\mathbf{Q}}{dt} = G(\mathbf{P},\mathbf{Q})$, $G(\mathbf{P},0) = 0$, where $\mathbf{P}$ denotes the non-disease classes and $\mathbf{Q}$ is the disease classes of the model. Let $U_0 = (\mathbf{P}^*,0)$ be the fixed point of the model, then U_0 is globally asymptotically stable (g.a.s) if and only if $\mathcal{R}_0 < 1$ and the following conditions are satisfied

C_1 : $\frac{d\mathbf{P}}{dt} = F(\mathbf{P},0)$, $\mathbf{P}^*$ is globally asymptotically stable.
C_2 : $G(\mathbf{P},\mathbf{Q}) = Z\mathbf{Q} - \widetilde{G}(\mathbf{P},\mathbf{Q})$, $\widetilde{G}(\mathbf{P},\mathbf{Q}) \geq 0$ for $(\mathbf{P},\mathbf{Q}) \in \Omega_n$ where $Z = \frac{\partial G}{\partial \mathbf{Q}} U_0$.

Proof

From the model system (2) – (7), we find that $\mathbf{P} = (S,R)$ and $\mathbf{Q} = (E_A, E, T, I)^T$. The DFE of the model is $U_0 = (\mathbf{P}^*,0) = \left(\frac{\Lambda}{\mu},0,0,0,0,0\right)$ as defined by (12). The point $U_0 = (\mathbf{P}^*,0)$ is g.a.s if $\mathcal{R}_0 < 1$, thus $\frac{d\mathbf{P}}{dt} = F(\mathbf{P},0) = [\Lambda - \mu S, 0]^T$ hence condition C_1 is easily satisfied. For condition C_2

$$ZQ = \begin{pmatrix} -(\alpha_1+\mu) & 0 & 0 & 0 \\ 0 & -(\alpha_2+\mu) & 0 & 0 \\ \alpha_1 & \alpha_2\rho & -(\psi+\phi+\mu) & 0 \\ 0 & \alpha_2(1-\rho) & \psi & -(\lambda+\mu) \end{pmatrix} \begin{pmatrix} E_A \\ E \\ T \\ I \end{pmatrix} \quad (1)$$

and

$$G(\mathbf{P},\mathbf{Q}) = \begin{pmatrix} \beta\varepsilon IS - (\alpha_1+\mu)E_A \\ \beta(1-\varepsilon)IS - (\alpha_2+\mu)E \\ \alpha_1 E_A + \alpha_2\rho E - (\psi+\phi+\mu)T \\ \psi T + \alpha_2(1-\rho)E - (\lambda+\mu)I \end{pmatrix}. \quad (2)$$

From the identity that $G(\mathbf{P},\mathbf{Q}) = Z\mathbf{Q} - \widetilde{G}(\mathbf{P},\mathbf{Q})$, then it implies using (1) and (2) we have

$$\widetilde{G}(\mathbf{P},\mathbf{Q}) = \begin{pmatrix} \beta\varepsilon I\left(1-\frac{S}{N}\right) \\ \beta(1-\varepsilon)I\left(1-\frac{S}{N}\right) \\ 0 \\ 0 \end{pmatrix} \quad (3)$$

where $\widetilde{G}(\mathbf{P},\mathbf{Q}) \geq 0$ if $\left(1-\frac{S}{N}\right) \geq 0$. We note that since, $S \leq N$ then $\frac{S}{N} \leq 1$ and hence $\left(1-\frac{S}{N}\right) \geq 0$. Further more, all the model parameters and the state variables are defined to be positive and thus $\widetilde{G}(\mathbf{P},\mathbf{Q}) \geq 0$ holds for the model satisfying the condition C_2. Based on the proof, we conclude that the DFE of the model is globally asymptotically stable whenever $\mathcal{R}_0 < 1$.

2. Labeled contour plots for sensitivity analysis

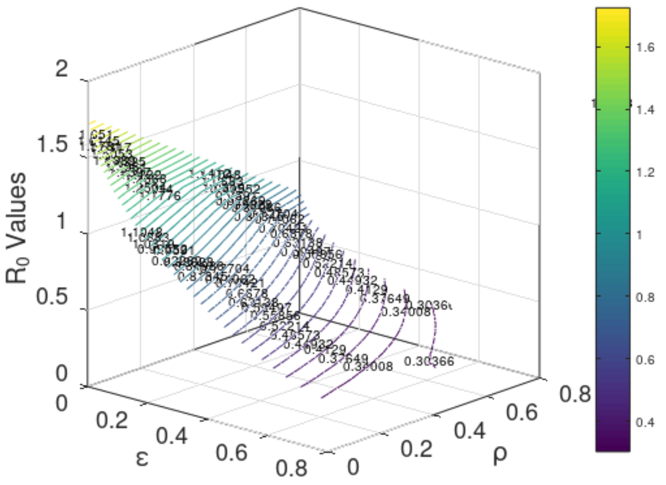

Figure S1. Labeled Contour plot visualization for Figure 3.

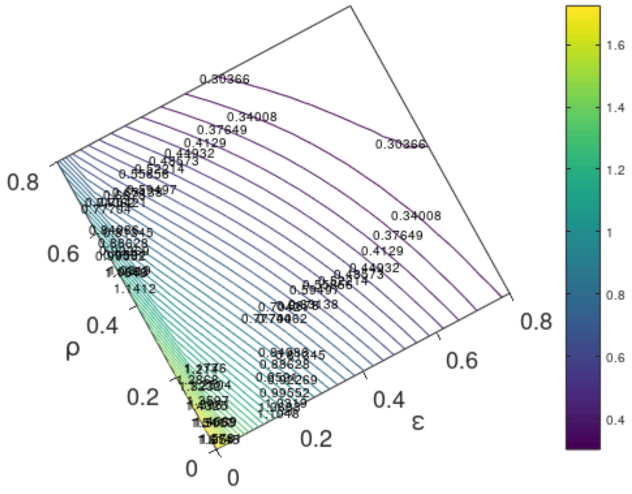

Figure S2. Labeled Contour plot visualization for Figure 3.

AIMS Press

©2022 the Author(s), licensee AIMS Press. This is an open access article distributed under the terms of the Creative Commons Attribution License (http://creativecommons.org/licenses/by/4.0)
